# Phylogeographic patterns in the Philippine archipelago influence symbiont diversity in the bobtail squid*–Vibrio* mutualism

**DOI:** 10.1002/ece3.4266

**Published:** 2018-07-02

**Authors:** Randy L. Coryell, Kira E. Turnham, Evelyn G. de Jesus Ayson, Celia Lavilla‐Pltogo, Angel C. Alcala, Filippina Sotto, Benjamin Gonzales, Michele K. Nishiguchi

**Affiliations:** ^1^ Department of Biology New Mexico State University Las Cruces New Mexico; ^2^ Southeast Asian Fisheries Development Center (SEAFDEC) Iloilo Philippines; ^3^ Silliman University Dumaguete City Philippines; ^4^ University of San Carlos Cebu City Philippines; ^5^ Western Philippine University San Juan Philippines

**Keywords:** phylogeography, squid, symbiosis, *Vibrio*

## Abstract

Marine microbes encounter a myriad of biotic and abiotic factors that can impact fitness by limiting their range and capacity to move between habitats. This is especially true for environmentally transmitted bacteria that cycle between their hosts and the surrounding habitat. As geologic history, biogeography, and other factors such as water temperature, salinity, and physical barriers can inhibit bacterial movement to novel environments, we chose to examine the genetic architecture of *Euprymna albatrossae* (Mollusca: Cephalopoda) and their *Vibrio fischeri* symbionts in the Philippine archipelago using a combined phylogeographic approach. Eleven separate sites in the Philippine islands were examined using haplotype estimates that were examined via nested clade analysis to determine the relationship between *E. albatrossae* and *V. fischeri* populations and their geographic location. Identical analyses of molecular variance (AMOVA) were used to estimate variation within and between populations for host and symbiont genetic data. Host animals demonstrated a significant amount of variation within island groups, while symbiont variation was found within individual populations. Nested clade phylogenetic analysis revealed that hosts and symbionts may have colonized this area at different times, with a sudden change in habitat. Additionally, host data indicate restricted gene flow, whereas symbionts show range expansion, followed by periodic restriction to genetic flow. These differences between host and symbiont networks indicate that factors “outside the squid” influence distribution of Philippine *V. fischeri*. Our results shed light on how geography and changing environmental factors can impact marine symbiotic associations at both local and global scales.

## 1 INTRODUCTION

The dispersal of marine species across suitable habitats can be affected by physical barriers (temperature, distances across oceans, island formations) as well as life history strategies (e.g., dispersal method of larvae and adult motility; (Kool, Paris, Barber & Cowen, 2011). Biogeographic barriers, as reported by floral and faunal separations, occur worldwide and provide an opportunity to study how physical barriers coupled with other abiotic factors may be affecting species dispersal and ultimately distribution (Lohman et al., 2011; Tonon et al., 2015). Analysis of population structure and physical orientation of the distribution of taxa across these barriers has given us clues to the factors that fragment available habitat (Esselstyn et al., 2010). Although previous work has provided evidence for several causes for speciation among closely related populations in areas where distinct barriers exist, there is less known about species that coexist with one another and whether rules that govern distribution patterns via allopatric speciation influence such associations (Hellberg, Burton, Neigel & Palumbi, 2002; Palumbi, 1994).

One region that has been studied extensively for its unique patterns of biogeography and geologic history is the Indo‐Pacific barrier (IPB), which was created by the uprising of the Indonesian archipelago separating the Indian and Pacific oceans (Gaither, Toonen, Robertson, Planes & Bowen, 2010). Interestingly, dispersal mechanisms and rapid adult motility have allowed certain taxa in the region to cross the IPB due to various dispersal strategies and larval residence time prior to metamorphosis compared to other taxa which are geographically restricted (Horne, 2014; Liu, Chang, Borsa, Chen & Dai, 2014; Sorenson, Allen, Erdmann, Dai & Liu, 2014). As part of the IPB, the Philippine island archipelago is a “hotspot” for species diversity and endemism and has warranted investigation of the distribution of taxa across the region (Roberts et al., 2002). In the Philippines, the current research has focused on the phylogeographic distribution of some fishes, bent‐toed geckoes, as well as bivalves across established biogeographic margins that limit some other terrestrial and marine taxa (Carpenter & Springer, 2005; Esselstyn et al., 2010; Gaither & Rocha, 2013; Huxley, 1868; Lemer et al., 2016; Siler, Oaks, Esselstyn, Diesmos & Brown, 2010; Wallace, 1860, 1863). Local analysis of the distribution and connectivity of some marine taxa across the Philippines has also been investigated in western populations of the sea star *Linckia laevigata* and the giant clam *Tridacna crocea* near the island of Palawan, as well in the western portion of the Central Visayas (Alcazar & Kochzius, 2016; Juinio‐Menez, Magsino, Ravago‐Gotanco & Yu, 2003; Magsino, Ravago & Juinio‐Menez, 2002; Ravago‐Gotanco, Magsino & Juinio‐Menez, 2007). Interestingly, very few studies have examined the connectivity of populations across the whole of the Philippine archipelago and what impact physical factors, life history, and geographic barriers have on the distribution of mutualist partners. This has created a void in the knowledge of how local assemblages of mutualist associations are impacted by geography in this unique area. Therefore, there is a need to better understand whether the physical barriers in this area have shaped the distribution of coexisting marine organisms and to determine what impact these physical factors have on species interactions, particularly between microbes and their eukaryotic hosts.

Across the globe, sepiolid squids (Cephalopoda: Sepiolidae) form mutualistic associations with bioluminescent bacteria from the genera *Vibrio* and *Photobacterium* (γ‐Proteobacteria: Vibrionaceae (Herring, 1977). *Vibrio* bacteria are housed within a specialized internal organ called the light organ (LO), where the host provides a nutrient‐rich habitat for the symbiont, and in return, *Vibrio* bacteria provide luminescence to the squid to be used in a behavior termed counterillumination (Jones & Nishiguchi, 2004). Squid hosts use the *Vibrio*‐produced light to reduce their silhouette during the evening, which enhances their survivability and predation success (McFall‐Ngai, Heath‐Heckman, Gillette, Peyer & Harvie, 2012). After each nightly foraging session, approximately 95% of the *Vibrio* bacteria are vented out into the surrounding seawater, seeding the local area with symbiotically viable Vibrios (Boettcher, Ruby & McFall‐Ngai, 1996). Local cycling of symbiotic *V. fischeri* exposes these bacteria to a wide range of abiotic and biotic factors outside the host that can affect their fitness and ability to infect new hosts. This also allows for symbiotically competent free‐living bacteria to migrate to new host habitats, where they can invade and colonize different populations of sepiolids (Nyholm, 2004; Nyholm & Nishiguchi, 2008; Nyholm, Stabb, Ruby & McFall‐Ngai, 2000).

Earlier work on sepiolid squids has focused on the influence of geographic distance on symbiont prevalence and genotype in both sympatric and allopatric populations (Jones, Lopez, Huttenburg & Nishiguchi, 2006; Kimbell, McFall‐Ngai & Roderick, 2002; Zamborsky & Nishiguchi, 2011). Allopatric and sympatric populations for both squids and *Vibrio* bacteria show distinct population breaks that are not necessarily driven by host specificity. Additionally, host‐mediated factors along with abiotic variables such as water temperature and salinity have been known to shape these mutualist assemblages (McFall‐Ngai, 2014; McFall‐Ngai et al., 2012; Nishiguchi, 2000; Soto, Gutierrez, Remmenga & Nishiguchi, 2009). Collectively, either genomic comparison of closely related populations (Bongrand et al., 2016) or haplotype comparisons of allopatric populations of Indo‐west Pacific squid and their vibrio symbionts (Jones et al., 2006) do not address the connectivity of populations across physical and biogeographic barriers like those in the Philippines or across the IPB. Therefore, we examined the genetic architecture of *Euprymna albatrossae* (Cephalopoda: Sepiolidae) and their *V. fischeri* symbionts in the Philippine archipelago using a combined phylogeographic approach to determine whether host specificity or geographic location influence the distribution of symbiotic Vibrios in this region. The unique geographic origin of the Philippines, its proximity to deeper and colder water, as well as currents that move through the area allow for the investigation of what roles geography and host specificity have in the distribution of mutualistic associations.

## 2 METHODS

### 2.1 Specimen collection and bacterial isolation

**Figure 1:**
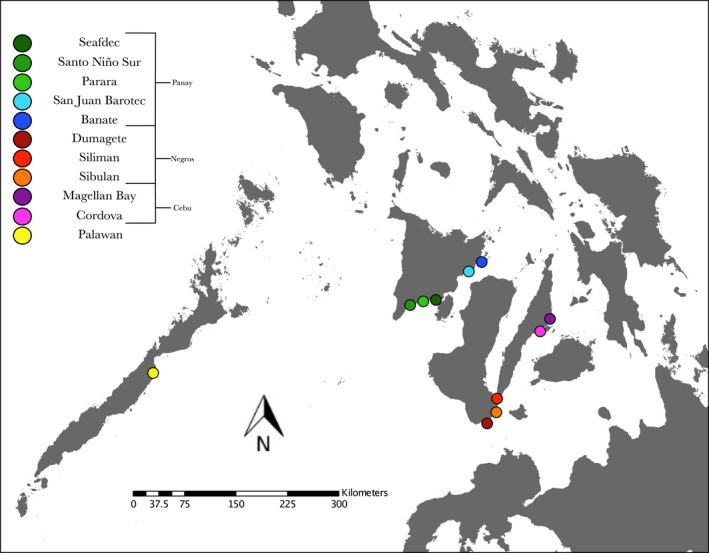
Sampling location where host squid were collected during the months of May, June, July, and August of 2010, 2012, 2013, and 2015

Squids were collected in the months May, June, July, and August during the years 2010, 2012, 2013, and 2015 at eleven different sites around the Philippine islands (Figure 1, Table 1). Adult squid (~2–4 cm in mantle length) were acquired either by dip or by seine net. Captured squids were brought back to the laboratory and placed on ice to anesthetize them prior to dissection. Host light organs were subsequently removed via ventral dissection and homogenized to plate on seawater tryptone agar plates (SWT; 0.5% tryptone, 0.3% yeast extract, 0.3% glycerol, 1.5% agar, and 70% seawater at 32 ppt) to isolate single colonies of *V. fischeri*. Plates used for light organ isolation were made with local seawater from SEAFDEC, while all other plates were made with artificial seawater containing a mixture of Instant Ocean (21 g/L of seawater; Spectrum Brands, VA) and Marine Mix (7 g/L of seawater; Wiegandt GmbH, Germany). Squid tissues were preserved in 95% ethanol for fixation and subsequent DNA extraction for Sanger sequence analysis at New Mexico State University (NMSU).

**Table 1:** Geographic location, sampling years, and sites where *Euprymna albatrossae* and *Vibrio fischeri* were collected in the Philippines during the months of May, June, July, and August of the years listed below

					Θ	
Site name	Abbreviation	Years collected	Host n	Coordinates (decimal, degrees)	Host	*Vibrio*
Cordova, Mactan	CVCP	2015	9	124.004, 10.317	0.00135	0.0051
Magellan Bay, Mactan	MGCP	2015	10	124.009, 10.528	0.0138	0.0046
Dumaguete, Negros	DBNP	2013	2	123.269, 9.186	0.1444	0.0074
Sibulan, Negros	SIBNP	2013	8	123.276, 9.396	0.0248	0.0061
Siliman, Negros	SBNP	2013	5	123.310, 9.31	0.0885	0.0050
Puerto Bay, Palawan	PBPP	2013, 2015	9	118.733, 9.733	0.0431	0.0048
Atabayan, Panay (SEAFDEC)	SEDEC	2010, 2012, 2013	8	122.400, 10.667	0.0055	0.0085
Banate, Panay	BAN	2012	10	122.815, 10.983	0.0067	0.0040
San Juan Barotac, Panay	SJBV	2012	7	122.872, 10.027	0.0467	0.0058
Santo Nino Sur, Panay	SNSP	2015	7	122.504, 10.679	0.0441	0.0052
Parara, Panay	PARA	2013	6	122.353, 10.700	0.0722	0.0076

*Note*

All squids were wild‐caught adults and approximately 2‐4 cm in mantle length.

Light organ homogenates were grown for 12 to 24 hr at 20°–28°C, after which 10–15 individual colonies from each plate were stab‐inoculated into vials containing SWT agar and sealed for transport back to NMSU. After transport, each sample was recultured on SWT agar plates at 28°C for 12–24 hr. Single colonies were isolated and cultured in liquid SWT at 28°C and shaken at 225 rpm for 12–18 hr in an Innova 43 shaking incubator (New Brunswick Scientific, NJ). Each overnight culture was subcultured and allowed to reach log phase (2–3 hr at 28°C and 225 rpm), and the log‐phase cultures were used for DNA extraction and also frozen in 40% glycerol for storage at −80°C.

### 2.2 DNA extraction and amplification

*Euprymna albatrossae* DNA was extracted using approximately 25 mg of ethanol preserved tissue that was dissected from the gill or mantle of each squid. Dissected tissues were washed with 100 μL of nuclease‐free water to remove any residual ethanol. *E. albatrossae* DNA was extracted using the DNeasy© blood and tissue protocol for animal tissues (Qiagen, Valencia, CA). All genomic DNA extractions were visualized on a 1% agarose gel and quantified using a Nanodrop 9600 (ThermoFisher Scientific, Waltham, MA). Total DNA extracted from each individual squid sample was used to amplify a 658‐bp fragment of the cytochrome *c* oxidase subunit I (COI, Table 2; (Folmer, Black, Hoeh, Lutz & Vrijenhoek, 1994; Jones et al., 2006; Zamborsky & Nishiguchi, 2011). The cytochrome c oxidase subunit I gene has been shown to be highly conserved, at least at the amino acid level, across invertebrate taxa (Folmer et al., 1994; Jacobs & Grimes, 1986) and has been used extensively to elucidate population structure (Calderon, Garrabou & Aurelle, 2006; Lessios, Kane & Evolution, 2003; Palumbi, Grabowsky, Duda, Geyer & Tachino, 1997).

**Table 2:** Primer names, sequence, and sequence source used in the amplification of the glyceraldehyde phosphate dehydrogenase (*gapA*) locus from *Vibrio fischeri* and the cytochrome *c* oxidase subunit 1 (COI) locus from *Euprymna albatrossae* collected in the Philippines from 2010, 2012, 2013, and 2015

Primer name	Fragment size	Primer sequence	Source
*gapAF*	889 bp	5′‐GGATTTGGCCGCATCGGCCG‐3′	Jones et al. (2006) and Zamborsky and Nishiguchi (2011)
*gapAR*		5′‐GGATTTGGCCGCATCGGCCG‐3′	
COI F	658 bp	5′‐TAAACTTCAGGGTGACCAAAAAATCA‐3′	Jones et al. (2006) and Nishiguchi et al. (2004)
COI R		5′‐GGTCAACAAATCATAAAGATATTGG‐3′	

Isolation of DNA from *V. fischeri* light organ isolates was completed using the Qiagen DNeasy^©^ blood and tissue kit (Valencia, CA) Gram‐negative bacterial protocol. Approximately 2 × 10^9^ cells were transferred from each log‐phase culture to the extraction tube for centrifugation. After, the remaining pellet was used for extraction using the Qiagen protocol. Purified *V. fischeri* DNA was visualized on a 1% agarose gel and quantified using a Nanodrop 9600 (ThermoFisher Scientific, Waltham, MA). Isolated DNA extracted from each *V. fischeri* isolate was used to amplify a portion of the glyceraldehyde phosphate dehydrogenase (*gapA*) locus (~900 bp) by PCR, using previously described *Vibrio*‐specific primers (Table 2; (Jones et al., 2006; Nishiguchi & Nair, 2003). The *gapA* locus has been used reliably to estimate deep phylogenetic connections between bacterial families (Nelson, Whittam & Selander, 1991) within the Vibrionaceae (Thompson, Gomez‐Gil, Vasconcelos & Sawabe, 2007) as well local population structure of mutualist *V. fischeri* (Jones et al., 2006; Nishiguchi & Nair, 2003).

Each PCR amplification reaction (25 μL) contained 2–20 ng of template DNA [0.08–0.8 ng/μL], GoTaq DNA polymerase [0.05 U/μL] (Promega, Fitchburg, WI), 5× GoTaq buffer [1×] (Promega, Fitchburg, WI), a 10 mM deoxynucleoside triphosphate mix [0.8 mM] of each nucleotide (Promega, Fitchburg, WI), and both forward and reverse primers [0.5 μM each] (Table 2). All amplification reactions were run using a MJ Research Dyad Disciple thermocycler (Waltham, MA). Cycle conditions for each reaction are listed in Table 3. Amplicons were purified using QIAquick PCR purification kit (Qiagen, Valencia, CA) and quantified using a Nanodrop 9600 (ThermoFisher Scientific, Waltham, MA). Purified amplicons were presequenced using BigDye Terminator v3.1 (Applied Biosystems, Foster City, CA) and amplified on an MJ Research Dyad Disciple thermocycler (Waltham, MA). Presequencing samples were cleaned using 96‐well Sephadex plates (Edge Biosystems, St. Louis, MO). Samples were sequenced at the NMSU Molecular Biology Sequencing facility using the Applied Biosystems 3130XL sequencer (Applied Biosystems, Foster City, CA). Sequences were assembled and aligned using GENEious (Biomatters Ltd, v7).

**Table 3:** Results of identical AMOVA performed on host and symbiont genetic data

Source of *E. albatrossae* variation	*df*	Sum of squares	Variance components	Percentage of variation
Among islands	3	1340.934	23.8035^a^	66.61
Within islands	7	143.175	1.4918	4.29
Within populations	70	709.212	10.1316^a^	29.10
Total	80	2193.321	34.8127	
Overall (*F*_ST_)	0.70897^a^			

Source of *V. fischeri* Variation	*df*	Sum of squares	Variance components	Percentage of variation
Among islands	3	57.057	0.1799	5.52
Within islands	7	66.639	0.4693^a^	14.40
Within populations	168	438.538	2.6704^a^	80.08
Total	178	562.235	3.2596	
Overall (*F*_ST_)	0.1991^a^			

*Note*

a *p* < 0.001.

### 2.3 Haplotype networks, nested clade analysis, and molecular variance

Haplotype networks for squid and symbiont were generated using TCS v1.12 using statistical parsimony methods outlined by Templeton (Templeton & Sing, 1993). Nested clade analyses were performed using Templeton's nesting algorithm as implemented in GEODIS (Posada, Crandall & Templeton, 2000). Analysis of molecular variance (AMOVA) was executed using the population genetics software platform ARLEQUIN (Excoffier & Lischer, 2010). Analyses were run for measures of within‐ and among‐population variation along with a separate analysis assessing variation by island for both host and symbiont data. Concurrently, theta (Θ), a base‐pair‐by‐base‐pair measure of polymorphism was calculated for each mutualist population at each sample site.

## 3 RESULTS

### 3.1 Nested clade and molecular variance analysis of *E. albatrossae*

**Figure 2:**
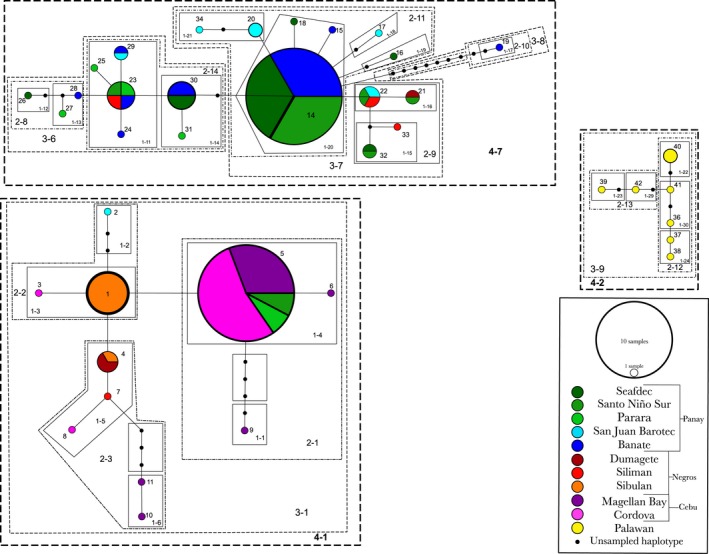
TCS nested haplotype network generated from *Euprymna albatrossae* molecular data acquired from animals captured in the Philippines during the years 2010–2015. Each line in the diagram represents one‐base‐pair mutational step between haplotypes. Black circles represent unsampled mutational steps connecting haplotypes. The size of each circle is indicative of the number of sequences that make up that haplotype, with the color of each circle representing the geographic origin of the sequence data and its proportion of the total haplotype. Each haplotype is represented by a two‐digit indicator with the dotted line enclosures indicating the nesting hierarchy. Each nesting level is labeled with a dashed two‐digit label

A total of 81 host COI sequences were used in the nested clade analyses of host genetic data, resulting in 43 distinct squid haplotypes (GenBank; MF379363–MF379405). Host genetic data yielded three distinct unconnected haplotype networks, with one network containing only samples from the island of Palawan, while the other two networks exhibited introgression from the central island chain but no connection from the Palawan population (Figure 2). Interestingly, host haplotype networks demonstrate little genetic connection between geographically separated populations. One host network (clade 4‐7, Figure 2) has most members of this haplotype from the island of Panay with small contributions from populations found near the island of Negros, but no contributions from nearby Cebu populations. Another separate host network (clade 4‐1, Figure 2) demonstrates genetic connection between Cebu, Negros, and a small contribution from Panay but also no connection to Palawan. The largest haplotype in clade 4‐7 (haplotype 14) is the result of equal contributions of genetic information from SEAFDEC, Santo Niño Sur, and Banate, all from the island of Panay. The dominant haplotype from clade 4‐1 (haplotype 5) has the largest contribution from populations sampled at Cordova and Magellan Bay from the island of Cebu, with a small contribution in this clade from SEAFDEC and Santo Niño Sur populations from the island of Panay. The third host network, clade 4‐2, has no connection between Palawan host populations and hosts from the central island chain (Figure 2).

Final nested clade analysis was performed with no detectable loops according to the rules established by Templeton (Templeton & Sing, 1993). Nested clade phylogenetic analysis of host genetic data demonstrates that the null hypothesis of panmixia was rejected in four of the nested clades and for the total cladogram (Figure 2). Inference from clade 2‐3 indicates allopatric fragmentation involving populations from Cebu and Negros (Table 4; Figure 2). Clade 2‐11 has restricted gene flow with isolation by distance involving populations from the island of Panay exclusively. Clade 3‐1 also demonstrated restricted gene flow with isolation by distance, with clade 2‐3 nested within and including genetic contributions from populations from all three central islands sampled (Table 4; Figure 2). Clade 3‐7, which includes subclade 2‐11 as an interior clade (Table 4), inferred contiguous range expansion for populations. Total host cladogram inference was inconclusive due to the lack of connection between higher level clades (Table 4).

**Table 4:** *Euprymna* clades that demonstrated significance during either a permutation contingency analysis or geographic distance analysis

Clade	Nested clade	Distance	Value (S or L)	*p*	Inference key steps	Inference
2‐3	1‐6`(T)	*D*_n_	102.87 S	0.0485	1,19, NO	Allopatric fragmentation
	1‐5 (I)	*D*_c_	23.288 S	0.0485		
		*D*_n_	38.528 S	0.0485		
	I‐T	*D*_n_	−64.3381 S	0.0485		
2‐11	1‐21 (T)	*D*_c_	0.0 S	0.0322	1,2,3,4, NO	Restricted gene flow with isolation by distance
3‐1	2‐1 (T)	*D*_c_	54.307 S	0.0094	1,2,3,4, NO	Restricted gene flow with isolation by distance
3‐7	2‐9 (T)	*D*_n_	97.411 L	0.048	1,2,11,12, NO	Contiguous range expansion
	2‐11 (I)	*D*_c_	31.471 S	0.0406		
		*D*_n_	61.135 S	0.0417		
	I‐T	*D*_c_	−57.4872 S	0.0386		
		*D*_n_	−36.2756 S	0.0428		
Total	4‐1	*D*_c_	75.7056 S	0.0002	1,2, IO	I‐T status undetermined: inconclusive outcome
	4‐2	*D*_c_	0.000	>0.001		
		*D*_n_	435.1782 L	>0.001		
	4‐7	*D*_c_	67.0744 S	>0.001		
		*D*_n_	82.2657 S			

*Notes*

Location of significance is indicated by (*D*_n_), nested clade distance, and/or (*D*_c_), the within‐clade distance. I‐T indicates the average distance between a tip clade and an interior clade. S or L indicates that the distance measure is significantly smaller or larger at the 5% inference level. Inference steps were performed using the automated inference key in GEODIS, part of the AneCA v1.2 population genetics analysis software platform (Posada et al., 2000).

Analysis of molecular variance of host genetic data revealed that a significant portion of the variance was detected among islands and within populations (66.61%; 29.10%, Table 3). Some of the highest amounts of within‐population genetic diversity, reported as theta, were observed at Dumaguete, Sibulan, and Siliman, which are all populations located from the island of Negros (0.1444, 0.0885, 0.0248; Table 1). Additionally, theta measures of populations at Parara, San Juan Barotec, and Santo Niño Sur from the island of Panay demonstrate significant within‐population diversity at these sites (0.0722, 0.0467, 0.0441; Table 1). Genetic diversity among populations near the island of Palawan was also observed to be a significant source of variation (0.0431, Table 1). The lowest amount of genetic diversity was detected at Cordova on the island of Cebu, as well as at SEAFDEC and Banate from the island of Panay (0.00135, 0.0055, 0.0067; Table 1).

### 3.2 Nested clade and molecular variance analysis of *V. fischeri*

**Figure 3:**
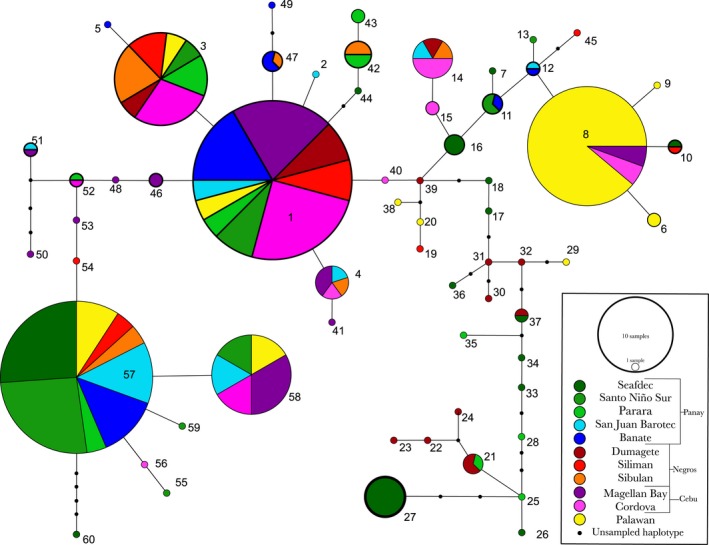
TCS haplotype network generated from *Vibrio fischeri* molecular data acquired from isolates harvested from squid light organs in the Philippines during the years 2010–2015. Each line in the diagram represents one‐base‐pair mutational step between haplotypes. Black circles represent unsampled haplotypes. The size of each circle is indicative of the number of sequences that make up that haplotype, with the color of each circle representing the geographic origin of the sequence data and its proportion of the total haplotype. Each haplotype is represented by a two‐digit indicator

**Figure 4:**
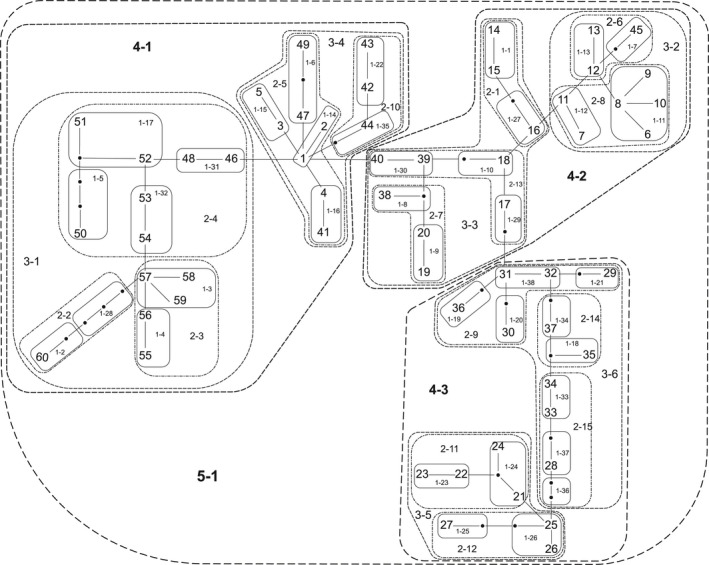
Nested *Vibrio fischeri* haplotype network generated from molecular data acquired in the Philippines during 2010, 2012, 2013, and 2015. Each haplotype is represented by a two‐digit identifier (see Figure 3), with each hierarchical nesting level represented by a two‐ to three‐digit dashed identifier and enclosed within dashed and dotted lines. Lines between haplotypes represent the mutational steps required to transition from one genetic station to another, with the small black dots representing unsampled haplotypes

Conversely, symbiont genetic architecture in the Philippines displays a different pattern compared to their host squid. Successful initial colonization of juvenile light organs is accomplished by 1‐3 strains that persist throughout the life of the animal (Wollenberg & Ruby, 2009). Any identical sequences, isolated from the same light organ, were removed. Analysis of 181 symbiont *gapA* sequences yielded one contiguous network of 60 haplotypes (Genbank; MF379406 – MF379465). In contrast to host genetic architecture, symbiont populations are more connected compared to host populations. *Vibrio* genetic data produced a highly connected and diverse network showing continuity between Palawan and the central island chain populations of symbionts (Figures 3 and 4). Haplotypes 1, 3, 8, 57, and 58 contain representatives from each of the island populations sampled (Figure 3). Major contributions from the western island of Palawan to haplotypes 8, 57, and 58 occur despite no host genetic introgression (Figures 2 and 3). The largest haplotype (haplotype 1) contains a significant number of Cebu haplotypes coupled with populations from Negros and Palawan. Each of the major haplotypes listed requires a minimum of one‐base‐pair change, with the largest number of changes needed (6) to go from haplotype 1 to haplotype 8.

Contingency analyses of symbiotic *V. fischeri* nesting revealed significant evidence for restricted gene flow with isolation by distance in clades 2‐1 and 2‐8 (Table 5). Clade 2‐1 exhibits restriction within the central island chain and connection between small haplotypes found near Panay, Negros, and Cebu (haplotypes 14, 15, and 16. Figure 4). Clade 2‐8, which includes haplotypes 6, 8, and 9 grouped together with haplotypes 7 and 11 (Figure 4), shows connection between central island populations and populations from Palawan. Inference from clade 3‐5 indicates continuous range expansion of these populations. The grouping of clade 3‐5 indicates a genetic connection between Panay and Dumaguete populations specifically from the island of Negros. Clade 4‐2 also illustrates continuous range expansion and includes the subclades 2‐1 and 2‐8, which at the lower nesting level demonstrate restricted gene flow and isolation by distance (Table 5). Clade 4‐2 also includes several singleton haplotypes that connect Palawan with the central island populations (Figure 4). Total cladogram inference indicates, as in some of the lower level clades, restricted gene flow with isolation by distance (Table 5).

**Table 5:** *Vibrio fischeri* clades that demonstrated significance during either a permutation contingency analysis or a geographic distance analysis

Clade	Nested clade	Distance	Value (S or L)	*P*	Inference key steps	Inference
2‐1	1‐1 (T)	*D*_c_	76.77 S	0.0218	1,19,20,2,3,4, NO	Restricted gene flow with isolation by distance (restricted dispersal by distance in nonsexual species)
		*D*_n_	84.88 S	0.0408		
	1‐27 (I)	*D*_n_	117.3675	0.0043		
	I‐T	*D*_c_	−76.7722	0.0667^NS^		
		*D*_n_	32.4858	0.0043		
2‐8	1‐11 (T)	*D*_c_	196.5289 S	0.0183	1,19,20,2,3,4, NO	Restricted gene flow with isolation by distance (restricted dispersal by distance in nonsexual species)
3‐5	2‐11 (T)	*D*_n_	99.7825 L	0.0139	1,2,3,4, NO	Contiguous range expansion
	2‐12 (I)	*D*_c_	0.833 S	0.0054		
		*D*_n_	73.3769 S	0.0139		
	I‐T	*D*_c_	−71.6474 S	0.0054		
		*D*_n_	−26.4056	0.0139		
4‐2	3‐2 (T)	*D*_c_	240.9458 L	0.0197	1,2,11,12, NO	Contiguous range expansion
		*D*_n_	241.0454 L	0.0045		
	3‐3 (I)	*D*_c_	104.4759 S	>0.001		
		*D*_n_	176.6458 S	0.0042		
	I‐T	*D*_c_	−136.4699 S	>0.001		
		*D*_n_	−64.3996 S	0.0041		
Total	4‐1 (T)	*D*_c_	104.559 S	>0.001	1,2,3,4, NO	Restricted gene flow with isolation by distance (restricted dispersal by distance in nonsexual species)
		*D*_n_	111.3004 S	>0.001		
	4‐3 (T)	*D*_c_	99.2733	0.0508*		
		*D*_n_	97.1363 S	0.0069		
	4‐2 (I)	*D*_c_	214.3525 L	>0.001		
		*D*_n_	198.3175 L	>0.001		
	I‐T	*D*_c_	110.8023 L	>0.001		
		*D*_n_	89.7202 L	>0.001		

*Notes*

Location of significance is indicated by (*D*_n_), nested clade distance, and/or (*D*_c_), the within‐clade distance. I‐T indicates the average distance between a tip clade and an interior clade. S or L indicates that the distance measure is significantly smaller or larger at the 5% inference level. Inference steps were performed using the automated inference key in GEODIS, part of the AneCA v1.2 population genetics analysis software platform (Posada et al., 2000). NS, not significant.

An identical AMOVA of symbiont genetic data revealed that a significant portion of the variance exhibited by these populations exists within and among populations (14.40%, 80.08%; Table 3). Base‐pair‐by‐base‐pair nucleotide diversity of host populations was highest in populations from Atabayan and Parara, Panay (0.0085, 0.0076; Table 1), and from the island of Negros (0.0074, 0.0061, 0.0050; Table 1), which are both in the central island chain. The lowest amount of genetic variation was observed at Magellan Bay, Mactan, and Banate, Panay (0.0046, 0.0040; Table 1); this was similar to what was detected in host diversity measures at these sites.

## 4 DISCUSSION

### 4.1 Host genetic architecture

The genetic structure of the *E. albatrossae* sampled for this study indicates that geographic location impacts host distribution. Island effects, as reported by the location and amount of variance during AMOVA, were detected in host genetic data, further supporting that geologic origin, physical geography, and possibly environmental factors have shaped the distribution of host squid in the region (among islands, *df* = 3, SS = 1340.934, VC = 23.8035, PV = 66.61%; Table 3). The genetic fixation observed in the host genetic data, reported as *F*_ST_, indicates that genetic flow is limited throughout the region and populations are genetically isolated from each other (*F*_ST_ = 0.70897, *p* < 0.0001; Table 3). This is reflected in the three distinct host networks detected in the Philippines (Figure 2) and suggests that geography may be influencing host genetic exchange and distribution. This could be because the benthic lifestyle adult *Euprymna* squid (~2–4 cm) lead as adults rarely migrate; however, the semipelagic nature of newly hatched squid (~3–5 mm) would allow water flow to transport juveniles to novel locations (Kimbell et al., 2002; Villanueva, Vidal, Fernández‐Álvarez & Nabhitabhata, 2016). Two of the distinct networks occur in the central island chain and, while having similar geologic origin, show no genetic connection in habitats that are homogeneous (Allen & Werner, 2002). Clade 4‐7 is distinctly made up of mostly squid haplotypes detected around the island of Panay, with small contributions from populations around Negros (haplotypes 21, 22, 23, and 33, Figure 2). This indicates that populations may have been fragmented due to the result of geologic activity in the region, seasonal changes in currents, or even modern‐day commercial fishing management, which have all been shown to influence fragmentation of marine habitats in the area (Abesamis, Russ & Alcala, 2006; Huang, Wu, Zhao, Chen & Wang, 1997; Savina & White, 1986; Wyrtki, 1961; Zhou, Ru & Chen, 1995). Habitat fragmentation was also inferred within clades 2‐11 and 3‐1 (Table 4), where genetic data indicate restricted gene flow with isolation by distance. Clade 2‐11 (Figure 2), comprised solely of host haplotypes detected around Panay, is closely connected (in some cases, only one‐base‐pair difference between haplotypes; Figure 2) to haplotypes with relatively small genetic contributions from the island of Negros. This indicates that the physical geography of the central island chain may be restricting host movement and genetic exchange. Additionally, clade 3‐7 (Figure 2) inferred contiguous range expansion between populations sampled from the islands of Panay and Negros despite these populations being isolated geographically (Table 4), suggesting that the alternating direction of prevailing currents in the region is one mechanism of dispersal as well as isolation for these squids.

**Figure 5:**
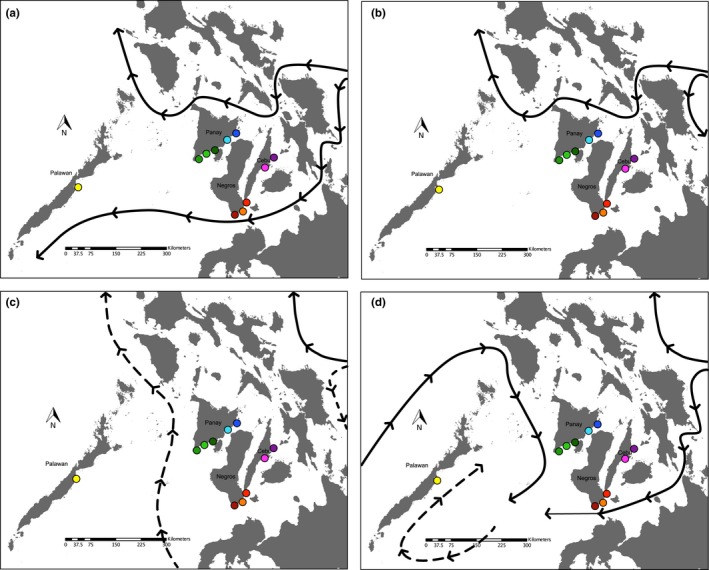
Maps of surface currents in the Philippine islands during (a) winter and early spring (December–February), (b) late spring (March–May), (c) early summer (June–July), and (d) late summer/fall (August–November). Adapted from Wyrtki, 1961

The divergence of the equatorial current (EC) as it approaches the Philippines from the east influences the directional flow of water through the central island chain, particularly north of the island of Panay and south of the islands of Negros and Cebu (Figure 5; (Huang et al., 1997; Wyrtki, 1961). The amount and speed of water that is funneled around or through the central islands depends on the time of year and is reflected in the patterns detected in host haplotype networks (Wyrtki, 1961). In late winter/early spring, water flows from the east to the west from the San Bernardino Strait, the south of Masbate, and finally around the north of Panay toward the Sulu Sea (Figure 5a). This flow pattern coupled with the flow of the southern divergence of the EC allows for genetic exchange between geographically isolated populations from Panay, Cebu, and Negros (Figure 2; clades 4‐1 and 4‐7) by allowing squid to be transported to areas they could not reach by themselves. As spring continues, the divergence pattern changes, and only a moderate east‐to‐west flow of water north of Panay is produced, while the bulk of the southern divergence is shifted south of the island of Mindanao, temporarily isolating squid populations south of Negros and Cebu from populations to the north of Panay (Figure 5b). During the summer months (June–August), waters within the central island chain are relatively still, with most of the equatorial current diverted northeast of Luzon and southeast of Mindanao, circling around to the north, into the Sulu Sea (Figure 5c). This isolates the central islands from the western island of Palawan and further prohibits exchange across the Sulu Sea. The two predominant haplotypes detected from our squid data (haplotypes 1 and 5; Figure 2) are separated by only one‐base‐pair difference even though the populations that contribute to these haplotypes are separated by physical barriers. This further supports the notion that currents may be influencing the prevalence and direction of gene flow between Panay squid populations and other squid assemblages to the south.

While clade 4‐1 had no valid inference (Table 4), clade 3‐1, which is fully nested within 4‐1 (Figure 2), had an inference of restricted gene flow with isolation by distance. This indicates that these habitats may have changed over time and influenced the genetic flow between once connected populations of host squid. A third separate network (clade 4‐2, Figure 2) consisted solely of samples collected around the western island of Palawan provides evidence that the unique geologic origin of Palawan may have fragmented a once continuous population of host animals (Sathiamurthy & Voris, 2006; Wallace, 1863; Zhou et al., 1995). Likewise, factors such as the deep‐water thermocline in the middle of the Sulu Sea and changes in surface water transport (Figure 5d) may have isolated these populations from others in the central islands (Chen, Yeh, Chen & Huang, 2015; Huang et al., 1997; Miao, Thunell & Anderson, 1994; Stuecker, Timmermann, Jin, McGregor & Ren, 2013).

### 4.2 Symbiont genetic architecture

Symbiont genetic data indicate that *Vibrio* bacteria seem to be able to mitigate the barriers that restrict host genetic exchange. Analysis (AMOVA) of total symbiont genetic data reveals that most of the genetic variation observed lies within each population in contrast to the partitioning of variation from host genetic data (*df* = 186, SS = 438.538, VC = 2.6704, PV = 80.08%; Table 3). The level of genetic diversity of the total symbiont population in the region is also indicative of symbiont gene flow between populations of hosts that are isolated from one another (*F*_ST_ = 0.1991, Table 3). One contiguous haplotype network was detected in the symbiont genetic data (Figures 3 and 4) revealing several genetic connections between symbionts collected from host squid that are genetically and geographically isolated from one another (Figure 2). The predominant haplotypes found within symbiont genetic data (haplotypes 1, 3, 8, 57, and 58; Figure 3) are comprised of samples from all the islands, regardless of geologic origin or physical position. In contrast to the pattern detected in host data, symbiont genetic data display connections between the western island of Palawan and the central Islands to the east (haplotypes 1, 3, 8, 57, and 58; Figure 3). Interestingly, *Vibrio* haplotype 8 (Figure 3), consisting of samples primarily from Palawan, has only 6‐base‐pair changes from the more diverse haplotype 1 (Figure 3). The composition of these two haplotypes (8 and 1, Figure 3) is quite different and could be a result of multiple introgressions of these populations by neighboring and distant *V. fischeri* symbionts displaced from their native range. When including intermediate haplotypes from Cebu (haplotype 40, Figure 3) and Negros (haplotypes 16, 11, and 12; Figure 3), there is evidence for connection between symbiont populations where hosts are restricted. While geography appears to have little influence on the population structure of symbiotic *V. fischeri* in this region, oceanographic currents may be influencing the ability of bacteria to cross barriers that restrict hosts.

### 4.3 Geography, geologic history, and environmental conditions

Results from this study indicate that geography plays a role in host squid distribution, without demonstrating a significant influence on symbiont distribution. The disparity in these patterns may be a result of differences in dispersal methodology between mutualist partners; that is, host squid have a limited range as adults and rarely travel far from their birthplace due to the limited dispersal ability of direct developing, benthic hatchlings (Kimbell et al., 2002; Villanueva et al., 2016). Conversely, symbiotically viable *Vibrio* bacteria are cycled out of the host daily exposing them to environmental factors (i.e., currents) that allow for movement into novel areas where they are able to recruit into a new host. While bacteria alone cannot cross great expanses of ocean, the use of rafting has been shown to be an effective dispersal mechanism for marine bacteria like *V. fischeri* (Jones et al., 2006; Theil & Gutow, 2005). The ability for vibrios to cross great expanses of oceans has been previously reported in other marine bacteria and undoubtedly will allow symbiotically viable vibrios to be shuttled to new areas and novel hosts (González‐Escalona, Gavilan, Brown & Martinez‐Urtaza, 2015).

Prevailing currents in and around the central Philippine island chain vary in direction and magnitude seasonally (Wyrtki, 1961). As *E. albatrossae* breed all year long, this change in directionality may provide newly hatched squid the opportunity to be carried to new areas, despite their otherwise limited dispersal ability, while being cut off from other available habitats when the prevailing currents change (Hanlon, Claes, Ashcraft & Dunlap, 1997). The pattern of direction in the symbiont genetic data presented here indicates that introgression across the Sulu Sea, which appears to be a biogeographic margin for host animals, is facilitated by the directional flow of water during the monsoon season (Huang et al., 1997). *Euprymna* hatchlings are known to be “pelagic”; that is, they linger in the water column before settling to their benthic lifestyle (Moltschaniwskyj & Doherty, 1995). This also might heighten the ability of host populations to move to new localities.

Geologic changes and the physical oceanography of this region may also explain the patterns detected in the genetic data. Glacial maximum sea levels exposed portions of what was host native range within the central island chain, creating a disconnect between populations in the west and central island squid assemblages (Gaither & Rocha, 2013; Gordon, 2005; Zhou et al., 1995). During glacial norms, hosts are restricted by a deep‐water thermocline that has persisted since before the Holocene, between Palawan and the central island chain (Miao et al., 1994). Fluctuating sea level during glacial cycles as well as Cenozoic volcanic uprising of the central Visayas may also explain the disjunctive distribution of host animals across this region (Miao et al., 1994; Zhou et al., 1995). Given that many of the more abundant haplotypes examined have prevalence in localities that are geographically distinct, this provides additional evidence that host populations have been established well beyond the geologic history of the Philippines (e.g., Palawan).

While previous research has shown that symbiont gene flow can be restricted by temperature, symbionts in this region seem to be able to mitigate environmental barriers which hosts cannot, crossing geographic and biologic barriers with apparent ease (Nishiguchi, 2000). Symbiont gene flow demonstrates a current dependent directionality of introgression by vibrios from the central islands west to Palawan in the winter and west to east in the summer months (Huang et al., 1997). The El Niño Southern Oscillation has also been shown to influence not only sea surface temperatures, wind direction, and rainfall in this region, but also the position of this deep‐water thermocline, further isolating local populations of host squid while not restricting symbiont distribution (Chen et al., 2015; Stuecker et al., 2013). While other Indo‐west Pacific and Mediterranean populations of *Vibrio* demonstrate that some degree of host specificity, geography, or other environmental factors can impact symbiont genetic architecture, findings from this study indicate that geography alone cannot explain symbiont distribution and that physical factors (e.g., currents) are important drivers of microbial diversity in the region (Jones et al., 2006; Zamborsky & Nishiguchi, 2011).

Beneficial associations like the sepiolid squid–*Vibrio* mutualism will undoubtedly be impacted by the reduction of available habitat, highlighting the importance of investigating the influence geography has on symbiont prevalence and distribution. Findings from this study point to a need to better understand the mechanisms that will impact symbiotic associations across a changing landscape and what factors will influence the fitness of beneficial microbes when they are moved to a novel habitat. Our findings have provided clues as to how established populations of host squids are the foundation for symbiont population structure, yet abiotic factors still influence where vibrios can move and establish new populations.

## AUTHOR CONTRIBUTIONS

R.L. Coryell extracted DNA, amplified genes of interest, aligned and analyzed sequence data, and wrote the paper. K.E. Turnham collected samples, generated data, analyzed data, and cowrote the paper. E.G.J. Ayson and C. Lavilla‐PItogo coordinated collecting of sample animals as well as provided laboratory facilities for processing of animals and culturing of symbiotic bacteria. A. Alcala coordinated collecting of sample animals as well as provided laboratory facilities for processing of animals and culturing of symbiotic bacteria. F. Sotto coordinated collecting of sample animals as well as provided laboratory facilities for processing of animals and culturing of symbiotic bacteria. B. Gonzales coordinated collecting of sample animals as well as provided laboratory facilities for processing of animals and culturing of symbiotic bacteria. M.K. Nishiguchi coordinated and obtained project funding, initial experimental design, collection and processing of sample animals, coauthor of the paper, and contributed to editing and review of the manuscript.

## DATA ACCESSIBILITY

Final DNA sequences: GenBank accessions: *Euprymna albatrossae* (COI;MF379363—MF379405); *Vibrio fischeri* (*gapA*; MF379406—MF379465).
